# When It Comes to Electric Vehicle Emissions, Location Matters

**DOI:** 10.1289/ehp.120-a230a

**Published:** 2012-06-01

**Authors:** David C. Holzman

**Affiliations:** David C. Holzman writes on science, medicine, energy, economics, and cars from Lexington and Wellfleet, MA. His work has appeared in *Smithsonian*, *The Atlantic Monthly*, and the *Journal of the National Cancer Institute*.

In an effort to boost sales the Chinese government recently announced it would waive the 10% sales tax on domestic electric cars on top of government subsidies totaling up to $19,000 per car.^^1^^ But despite their green reputation, on a per-kilometer basis electric cars in China cause over 3.5 times more air pollution–related premature deaths than gasoline-powered cars, according to new estimates calculated for the country’s 34 largest cities.^^2^^

In the study, the researchers calculated emissions per person-km traveled—that is, a person traveling 1 km in a vehicle (if 15 people travel 10 km in a bus, the bus accumulates 150 person-km). They considered five vehicle types: electric cars (not including hybrids), electric bicycles and scooters (e-bikes), gasoline cars, diesel cars, and diesel buses. They estimated tailpipe emissions for gasoline and diesel vehicles based on emissions standards and figures published in the peer-reviewed literature. Electric vehicles do not produce combustion emissions themselves; instead, their emissions impact comes from the power plants that produce the electricity they use. The vast majority of electricity in China is coal-fired^^2^^ (compared with slightly less than 50% in the United States^^3^^).

The authors modeled the fraction of fine particulate matter (PM_2.5_) emissions inhaled by the population to estimate total attributable excess mortality. “This is the pollutant with the most well-studied health impacts, including irrefutable association with premature mortality,” says coauthor Christopher R. Cherry, an assistant professor in the University of Tennessee–Knoxville Department of Civil and Environmental Engineering.

**Figure:**
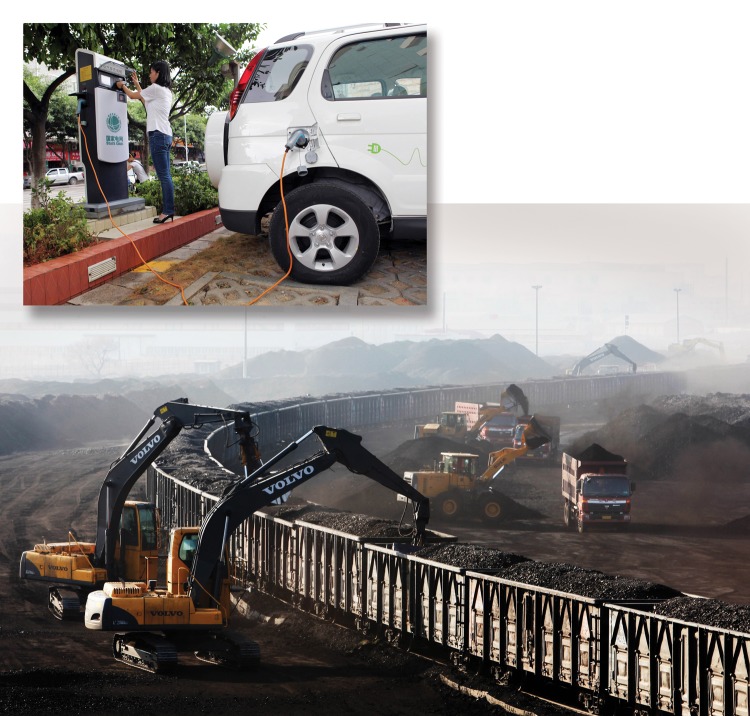
Electric vehicle emissions derive not from gasoline or diesel but from coal and other energy sources used to power the electricity grid. © Gao Jinxu/Xinhua Press/Corbis; Inset: © Imaginechina/Corbis

Although vehicle-specific mortality varied greatly from city to city, electric cars were estimated to cause more premature deaths than gasoline cars in 33 of the 34 cities surveyed.^^2^^ “Emissions from coal-fired power plants are comparatively high in China because of lower-quality coal and fewer plants using emission-control technologies,” explains report coauthor Julian D. Marshall, an assistant professor of environmental engineering at the University of Minnesota, Minneapolis.

In Shanghai, for instance, power-plant emissions associated with electric cars caused an estimated 26 excess deaths annually per 10 billion person-km versus 9 excess deaths for gasoline cars. Diesel cars caused an estimated 90 excess deaths per 10 billion person-km. Diesel buses, which have lower emissions per person-km than diesel cars because they carry more people, caused an estimated 32 excess deaths, and e-bikes performed the best, causing only 3 estimated excess deaths per 10 billion person-km per year.

Beijing exempts battery-electric vehicles from its monthly license-plate lottery to determine who is allowed to buy a new car. In March 2011 nearly 400,000 people vied for the monthly allotment of 17,600 license plates.^6^ In another effort to reduce traffic emissions, the city also occasionally uses odd/even rationing based on license plate number to control the number of cars on the road.^7^

Not surprisingly, the implications of this research for other countries are variable. Vietnam, for example, depends much more on natural gas and hydropower than coal for its electric power, says Cherry, such that electric cars there cause one-third as much pollution as gasoline cars and one-tenth as much pollution overall as they do in China. Conversely, he says, in India, average power-sector emissions of PM_2.5_ are 10% greater than in China, causing more pollution per kilometer per electric car.

“It is important to remember that electric vehicles are only as clean as the electricity that charges them, and a clean energy future includes both electric vehicles and a cleaner electricity grid,” says Don Anair, a senior analyst and engineer at the Union of Concerned Scientists (UCS). Anair notes that the U.S. grid is cleaner than China’s as a result of clean-air regulations and increased investment in renewable electricity (the U.S. investment in renewable energy ranks second in the world, just behind China^^4^^). The emissions intensity of the U.S. grid—that is, the emissions per unit of power produced—will continue to improve as older, unimproved coal-fired plants are retired, he says.

A report from the UCS released in April 2012 found that even in the U.S. region with the most emissions-intensive electricity grid, greenhouse-gas emissions attributable to electric vehicles are equivalent to those produced by gasoline cars that get 31–40 miles per gallon.^^5^^ That report did not address mortality attributable to PM_2.5_ exposure.
